# Frequency and risk factors of metabolic associated fatty liver disease among medical students in Egypt

**DOI:** 10.1038/s41598-025-95753-w

**Published:** 2025-04-18

**Authors:** Mohamed M. Elhoseeny, Fatma Rageh, Samar M. Rezk, Amira A. A. Othman

**Affiliations:** 1 Internal Medicine Department, Faculty of Medicine, Suez University, Suez, 43511 Egypt; 2 Infectious Diseases, Gastroenterology and Hepatology Department, Faculty of Medicine, Suez University, Suez, Egypt; 3 Clinical Nutrition Department, Mahalla Hepatology Teaching Hospital, El Mahalla El Kubra, Egypt

**Keywords:** FibroScan®, InBody 270, MAFLD, NAFLD, Steatosis, Transient elastography, Diseases, Gastroenterology, Health care

## Abstract

Metabolic (dysfunction) associated fatty liver disease (MAFLD) is a growing global concern. This study assessed the frequency of hepatic steatosis and MAFLD, alongside their associated risk factors, among medical students at Suez University, Egypt. A cross-sectional study was conducted from November 2022 to April 2023 among 84 medical students aged ≥ 18 years. Data on anthropometric parameters, body composition, and lifestyle were collected through self-administered questionnaires, InBody analysis, and FibroScan. MAFLD diagnosis required steatosis (≥ 238 dB/m) with obesity, metabolic dysfunction, or both. Statistical analyses included chi-square tests, ANOVA, and logistic regression. Hepatic steatosis was present in 25% of participants, while MAFLD frequency was 13.1%. Participants with MAFLD exhibited higher body weight (82.34 ± 10.78 kg vs. 65.84 ± 10.61 kg, *p* < 0.001), BMI (29.05 ± 3.66 vs. 22.90 ± 3.23 kg/m^2^, *p* < 0.001), waist circumference (88.73 ± 8.73 cm vs. 78.10 ± 7.96 cm, *p* < 0.001), BMR (1566.09 ± 27.37 vs. 1429.86 ± 93.44 kcal/day, *p* < 0.001), and fat mass (32.74 ± 7.25% vs. 23.91 ± 8.60%, *p* < 0.001). Binary regression analysis revealed increased body weight, BMI, waist circumference, and BMR as significant risk factors for MAFLD. An elevated fat mass percentage with a reduced muscle mass percentage highlighted the sarcopenic obesity role in MAFLD progression. Extreme weight reduction can exacerbate hepatic fat accumulation. Poor sleep quality, a sedentary lifestyle, and an unhealthy diet are also significant predictors. The widespread frequency of steatosis and MAFLD highlights the pressing need to tackle this silent epidemic among young Egyptian adults.

## Introduction

Metabolic (dysfunction) associated fatty liver disease (MAFLD), formerly named non-alcoholic fatty liver disease (NAFLD), is a group of clinical conditions characterized by the presence of hepatic steatosis with high risks of metabolic disorders. In contrast to NAFLD, MAFLD does not necessitate ruling out other liver disease causes, such as viral hepatitis or heavy alcohol use^1^. The new name chosen to replace NAFLD reflected the disease’s etiology better and conveyed a more practical approach in daily clinical practice than NAFLD, considering its close association with metabolic syndrome^2^.

The clinicopathological spectrum of MAFLD included simple fatty infiltration (≥ 5% hepatic steatosis), lipotoxicity, and inflammatory damage to hepatocytes causing metabolic dysfunction–associated steatohepatitis (MASH), progressed to hepatic fibrosis, and rarely advanced to lethal sequelae as cirrhosis, and hepatocellular cancer in about 30% to 40% of cases^3^. MAFLD is a serious challenge because of its high prevalence rate, lack of agreement on definitive diagnostic tools, difficulty identifying symptoms, complex etiology, limited awareness of the disease, and absence of approved treatments^4^. It can be a consequence of non-drinker obesity (BMI > 25 kg/m2 in white and > 23 kg/m2 in black) or type 2 diabetes mellitus (primary)^5^. It may result from a toxin or drug (secondary)^6^. Patients with fatty livers run the risk of developing diabetes, cardiovascular disease, renal disease, cirrhosis, and liver cancer, all of which have a major negative impact on the quality and health of their lives^7^.

The worldwide prevalence of MAFLD is about 25–30% of the global population. The highest rates were reported in the Middle East (32%), South America (31%), Asia (27%), the USA (24%), and Europe (23%), whereas the lowest rates were reported in Africa (14%)^8,9^. MAFLD prevalence in China was 21.18%^10^. The actual size of the MAFLD problem in Egypt is not fully recognized. MAFLD was the cause of 12.8% of cirrhosis-related deaths in Egypt in 2017, with other causes accounting for 6.5% of cases, most likely from undiagnosed MAFLD. Moreover, the age-standardized prevalence rates of MAFLD-related compensated and decompensated cirrhosis per 100,000 people rose from 312.3 and 19.4 in 1990 to 340 and 26 in 2017, respectively^11^. Studies reported mean and/or median age ranges from 30.7 to 76.2 years, reflecting the increasing prevalence of MAFLD with age^8^. Furthermore, people with metabolic syndrome (MetS), obesity, hypertension, type 2 diabetes (T2D), dyslipidemia, and hypertension are more likely to have MAFLD^12^. Patients with T2D are projected to have a global prevalence of 55.5% for MASLD^13^. It is anticipated that as obesity and diabetes rates rise globally, so will the prevalence of MASLD^14^.

Early diagnosis and management of fatty liver disease are essential prerequisites in safeguarding the health of the population and reducing the financial burden of national health, especially given the lack of sufficient knowledge about MAFLD among healthcare providers and patients^15^. Hepatic steatosis diagnosis in the National Health and Nutrition Examination Survey (NHANES) 2017–2018 in the USA and the Comprehensive Prevention Project for Overweight and Obese Adolescents (CPOOA) study in China is based on vibration-controlled transient elastography (VCTE) and abdominal ultrasonography, respectively. NHANES diagnosis was based on two measures of VCTE: median liver stiffness measurement (LSM ≥ 7.4 kPa) or median controlled attenuation parameter (CAP ≥ 248 dBm)^16^. Worth mentioning, that as the physicians select probes using BMI or the automated probe selection tool, the CAP and LSM accuracy and cut-off values diverge between different BMI populations and between different probes^17^. CPOOA diagnosis hepatic steatosis based on abdominal ultrasonography where at least two of the following must present: profound attenuation of the ultrasound signal, vascular blurring, and a diffusely enhanced echogenicity (or “bright”) liver with liver echogenicity greater than kidney or spleen^18^. MAFLD diagnosis is based on the presence of liver steatosis (detected by liver histology (golden standard), imaging, or noninvasive biomarkers), together with the presence of at least one of three criteria, which include (i) overweight or obesity , (ii) type 2 diabetes mellitus (T2DM), and (iii) clinical evidence of metabolic dysfunction (including hypercholesterolemia, hypertriglyceridemia, insulin resistance (IR), increased waist circumference, and/or systemic hypertension)^19^.

Our objective was to assess the frequency and risk factors of MAFLD among apparently healthy students at the Faculty of Medicine, Suez University. Given the specific study population, the findings reflect occurrence rather than true prevalence.

## Materials and methods

### Study design and setting

A prospective cross-sectional study was conducted at the faculty of medicine, Suez University, Suez, Egypt between November 2022 and April 2023. The study was conducted after obtaining approval from the local ethics committee, Faculty of Medicine, Suez University (Approval No. 32). Written informed consents were obtained from the included participants. All methods were carried out following relevant guidelines and regulations. It was performed according to the recommendations of Good Clinical Practice and the Declaration of Helsinki (2013).

This study employed a cross-sectional design to assess the frequency of MAFLD and its associated risk factors. While cross-sectional studies cannot establish causality, they are widely used in epidemiological research to identify patterns and associations in population health. The findings from this study provide a foundation for future longitudinal research that can assess temporal relationships and disease progression.

This study estimated the frequency of MAFLD among medical students at Suez University using a cross-sectional design. Since the sample was not randomly selected from the general population, the results should not be interpreted as true prevalence but rather as an estimate of frequency within this specific subgroup. Selection bias may be present due to the homogeneous nature of the study population, which may not fully represent the general adult population.

### Eligibility criteria

**Fig. 1:**
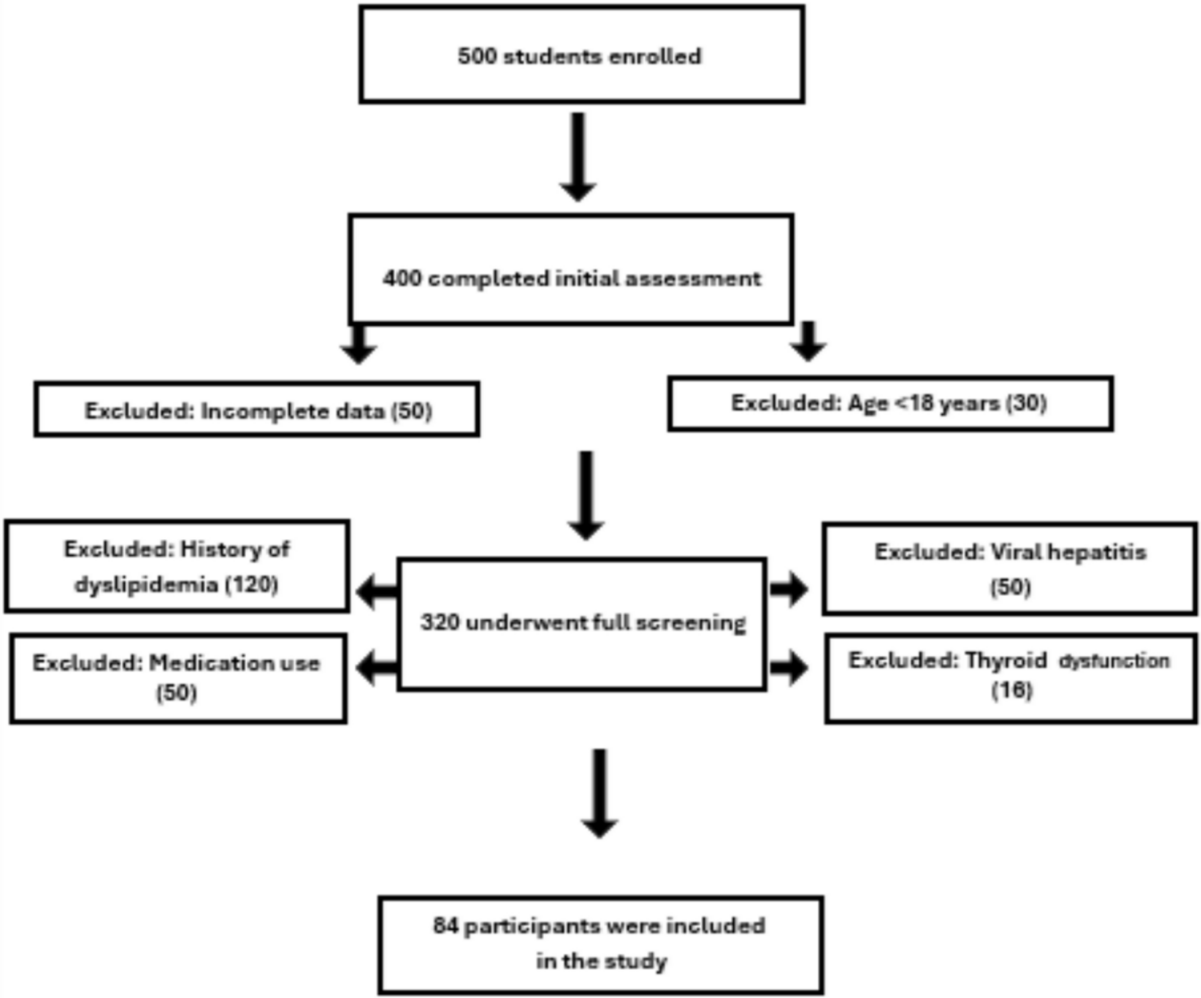
flowchart of participant selection and exclusion criteria.

This study included apparently healthy medical students of both sexes aged 18 years and older who voluntarily participated without financial compensation. Participants were required to have no prior history of liver disease or metabolic disorders requiring treatment. The final sample size of 84 participants was determined using OpenEpi version 3. Figure 1 provides a detailed flowchart of participant selection and exclusion criteria.

#### Inclusion criteria

Participants were eligible if they were medical students aged 18 years or older, in apparent good health, and willing to participate without financial compensation. Only individuals with no prior diagnosis of liver disease and no history of metabolic disorders requiring treatment were included.

#### Exclusion criteria

Exclusion criteria included individuals younger than 18 years and those diagnosed with viral hepatitis (HBV or HCV), chronic autoimmune hepatitis, Wilson’s disease, hemochromatosis, thyroid dysfunction, alpha-1 antitrypsin deficiency, or any other autoimmune disease. Participants with a history of dyslipidemia or a prior diagnosis of fatty liver disease were excluded. The study also excluded individuals using steatogenic medications such as amiodarone, valproic acid, tetracycline, antiretroviral therapy, or disease-modifying anti-rheumatic drugs (e.g., methotrexate). Participants receiving medications for MAFLD treatment, including vitamin E and thiazolidinediones, were also excluded. Additionally, individuals on metformin, glucagon-like peptide-1 (GLP-1) agonists, sodium-glucose cotransporter-2 (SGLT2) inhibitors, corticosteroids, antidepressants, or antipsychotic medications were not enrolled. Furthermore, participants with unreliable transient elastography results, defined as fewer than 10 valid measurements with an interquartile range/median (IQR/M) ratio > 30%, were excluded from the study.

## Data collection and study tools

### Clinical examination

All participants underwent a full medical history, family history, and social history taking with special considerations for age, sex, co-morbidities, and medications. A thorough, complete physical examination was also performed.

### Self-administered questionnaire (20)

Data were collected using a structured, self-administered questionnaire available in both Arabic and English. The questionnaire was designed to be completed in 10–15 min and covered sociodemographic characteristics, clinical history, dietary habits, physical activity, and sleep patterns. To ensure validity and reliability, dietary habits were assessed using a validated 32-item semi-quantitative Food Frequency Questionnaire (FFQ), standardized for the Egyptian population. Physical activity was evaluated using the International Physical Activity Questionnaire–Short Form (IPAQ), a widely validated tool, and sleep patterns were measured using the Pittsburgh Sleep Quality Index (PSQI), a validated instrument for assessing sleep quality and duration. The sociodemographic and clinical history sections were adapted from prior epidemiological studies and underwent expert review by hepatology and public health specialists. Additionally, a pilot study with 20 participants was conducted to assess clarity and feasibility, with internal consistency measured using Cronbach’s alpha (α = 0.78), indicating acceptable reliability.

Participants’ dietary habits were classified into two groups based on dietary patterns associated with liver fat accumulation. The “Healthy Eating” group was characterized by adherence to a Mediterranean diet, which included regular consumption of fruits, vegetables, whole grains (e.g., brown rice, oats), lean proteins (e.g., fish, chicken, legumes), and healthy fats (e.g., olive oil, nuts), with a moderate to low intake of sugars and processed foods. The “Unhealthy Eating” group followed a Western or pro-inflammatory diet, marked by a high intake of processed foods (e.g., fast food, ready-to-eat meals), sugary drinks (e.g., sodas, sweetened beverages), saturated and trans fats (e.g., fried foods, pastries, processed meats), low fruit and vegetable consumption, and excessive refined carbohydrates (e.g., white bread, pastries).

Participants were also categorized based on their physical activity levels. The “Active” group included individuals who engaged in regular physical activity, defined as exercising at least three times per week through activities such as walking, running, cycling, swimming, or strength training. The “Inactive or Sedentary” group included individuals who exercised fewer than three times per week or not at all and those who reported prolonged sitting periods or low overall daily physical activity levels.

Sleep patterns were classified into two groups based on sleep duration and quality, factors known to influence liver health. The “Healthy Sleep” group included participants who reported 7–9 h of sleep per night, good sleep quality, and minimal disturbances. The “Poor Sleep” group included individuals who slept less than 7 h per night, experienced frequent disturbances (e.g., insomnia, waking often), and reported poor sleep quality resulting in fatigue or feeling unrefreshed.

### Anthropometric measurements

Anthropometric measures were performed with the InBody 270 (InBody Co., Ltd., Seoul, South Korea), a portable bioelectrical impedance analysis (BIA) equipment noted for its ease and dependability in body composition studies. The InBody 270 measures critical variables such as body weight, skeletal muscle mass, body fat mass, body fat percentage, and visceral fat levels. This gadget combines an eight-point tactile electrode system and several frequencies to evaluate body composition with great precision. Unlike conventional BIA, which frequently changes readings based on age and gender, the InBody 270 calculates impedance for each individual, ensuring accuracy across various populations. Measurements were collected under established conditions to maintain consistency, such as fasting and minimal prior physical activity, which helps to reduce variability in impedance measurements due to recent meal or fluid intake. The portability and ease of use of the InBody 270 make it ideal for investigations that require repeated measurements in a variety of contexts. The device operates with an eight-point tactile electrode system and multiple frequencies, which improves the accuracy of measurements by directly assessing each segment^21^.

1. **Weight:** using a digital scale, the individuals’ body weight in light clothing and without shoes was determined and documented to the closest 0.5 kg^22^. In addition, the “InBody 270” gives a value for the various body compartments, which equals the weight of each compartment. When added together, they equal the person’s weight. The values can also be compared with the ‘normal scale’ values.
2. **Height:** the patient’s height was determined to the closest 0.1 cm while standing straight up against a wall and without shoes on^22^.
3. **Waist circumference:** using a tape measure while standing, the waist circumference was determined midway between the iliac crest and the final rib border. Normal waist circumferences (< 102 cm for men and < 88 cm for women), were typically used for identifying health risks^23^. The commonly used descriptive ranges in demographic or anthropometric studies for waist circumference are "Slim: < 85 cm," "Moderate: 85–95 cm," and "Broad: > 95 cm". Using this value with the InBody’s data (e.g., visceral fat levels and BMI) helps provide a better picture of central obesity and related health risks.
4. **Body mass index (BMI):** BMI was calculated from measurements of height (in meters) and weight (in kg) and expressed in kg/m^2^ using this formula weight [(kg)/ (height (m)]^2^. The cut-off points of “BMI of < 18.5 kg/m^2^, 18.5–24.9 kg/m^2^, 25.0–29.9 kg/m^2^, 30.0–34.9 kg/m^2^, 35.0–39.9 kg/m^2^ and 40.0 + kg/m^2^ define categories usually referred to as underweight, normal weight, overweight (pre-obese) and obese (grades I, II and III) respectively”^24^.

The InBody device calculates BMI using the entered height (in meters) and weight (in kilograms) and categorizes it according to the World Health Organization (WHO) standards. Individuals with a BMI < 18.5 are classified as underweight, those with a BMI of 18.5–24.9 fall within the normal weight range, BMI of 25–29.9 indicates overweight, and BMI ≥ 30 is categorized as obese

**Basal metabolic rate (BMR):**BMR is “the amount of energy needed while resting in a temperate environment when the digestive system is inactive”. The Harris-Benedict equations revised by the Mifflin-St Jeor equation in 1990 were used to calculate BMR^25^:

- For men: BMR = (10 × weight in kg) + (6.25 × height in cm) – (5 × age in years) + 5
- For women: BMR = (10 × weight in kg) + (6.25 × height in cm) – (5 × age in years) – 161.

The InBody device calculates BMR using the Mifflin-St Jeor equation, which considers age, gender, weight, and muscle mass. It integrates these parameters with bioelectrical impedance analysis (BIA) outputs to estimate daily energy expenditure at rest. BMR values typically range around 1,381 kcal/day for adult females and 1,744 kcal/day for adult males, with categorization based on individual body composition measurements.

5. **Muscle mass percentage**: The InBody 270 calculates muscle mass using segmental analysis, measuring impedance in different body regions (arms, legs, and trunk) to estimate skeletal muscle mass in kilograms. Muscle mass percentage is determined by dividing skeletal muscle mass by total body weight and multiplying by 100. This percentage represents the proportion of skeletal muscle relative to total body mass, making it a key metric in studies on muscle health, sarcopenia, and metabolic disorders ^26^. Optimal muscle mass percentage varies by sex and age group, with typical values ranging from 31–33% in adult females and 40–44% in adult males. Sarcopenia, characterized by a decline in muscle mass and strength, can result from aging, reduced physical activity, insufficient protein intake, or underlying comorbidities. It is reflected by a lower muscle mass percentage. In contrast, muscle hypertrophy refers to an increase in muscle size and strength, typically achieved through resistance training or targeted exercise, leading to an elevated muscle mass percentage^27^.
6. **Body fat percentage:** The InBody device measures the percentage of body fat (PBF) as a percentage of total body mass. Its software compares these values against population-specific reference ranges to classify body fat percentage as low, normal, or high, based on gender. Typical classifications include low (< 18%), normal (18–28%), and high (≥ 29%) for adult women, while for adult men, body fat percentages are categorized as low (< 10%), normal (10–20%), and high (≥ 21%)^27^.

### CAP and LSM measurements

Transient elastography (TE, FibroScan®) is a novel, non-invasive method for assessing liver steatosis and fibrosis with chronic liver disease, with immediate results and excellent reproducibility in the outpatient clinic. A FibroScan® 502 device, Echosens, Paris, France, equipped with the M (3.5 MHz) and XL (2.5 MHz) probes was used to assess both ***controlled attenuation parameter (CAP)*** and ***liver stiffness measurement (LSM)*** values.

Liver steatosis measured by CAP was expressed in decibels/meter (dB/m). CAP values range from “100 to 400 dB/m”. The following cut-off values were used for the diagnosis of steatosis stages: “Stage 0, < 238 dB/m, Stage 1, ≥ 238 to 260 dB/m, Stage 2, ≥ 260 to 292 dB/m, and Stage 3, ≥ 292 dB/m”. A median CAP of at least 238 dB/m indicates liver steatosis^28^. A healthy liver has a CAP score of 5% or lower. A CAP score between 238 to 260 dB/m represents 11–33% fatty change in the liver, between 260 to 292 dB/m represents 34–66% fatty change in the liver, and 292 dB/m or higher represents over 67% fatty change in the liver. Higher fat content is a risk factor for disease progression^29^. Liver fibrosis measured by LSM was expressed in kilopascal (kPa). LSM values range from “1.5 to 75 kPa”. A reliable LSM is obtained when having at least 10 valid measurements (a success rate of greater than 60%, and an interquartile range (IQR) < 30% of the median LSM value)^30^. The following cut-off values were used for the diagnosis of liver fibrosis stages: “no significant fibrosis or F0 < 6.2 kPa, mild fibrosis or F1 ≥ 6.2 to 7.6 kPa, moderate fibrosis or F2 ≥ 7.6 to 8.8 kPa, severe fibrosis or F3 ≥ 8.8 to 11.8 kPa, and cirrhosis or F4 ≥ 11.8 kPa”^31^.

MAFLD diagnosis was based on the FibroScan® device to diagnose hepatic steatosis in addition to the presence of one of the following three criteria: (i) overweight or obesity (BMI > 25 kg/m2), (ii) T2DM, and (iii) clinical evidence of metabolic dysfunction (including hypercholesterolemia, hypertriglyceridemia, insulin resistance (IR), increased waist circumference, and/or systemic hypertension).

### Statistical analysis

Data were analyzed using SPSS version 22 software (SPSS, Inc., Chicago, USA). Quantitative variables were expressed as mean ± standard deviation (SD), while qualitative data were expressed as numbers and percentages. Data normality was assessed using the Shapiro–Wilk test. Independent t-test was used to compare continuous variables between groups. Pearson’s Chi-Square test was used for categorical variables, with Fisher’s Exact Test applied when expected counts were < 5. Binary logistic regression analysis was performed to identify risk factors (predictors) for MAFLD. Odds ratios (ORs) and their 95% confidence intervals (CIs) were reported. In all cases, a *p*-value ≤ 0.05 was considered statistically significant.

## Results

### Socio-demographic and Anthropometry characteristics

The enrolled students’ (n = 84) ages ranged between 19 and 23 years, with a mean age of 21.02 ± 1.31 years. They had a nearly equal gender distribution (54.8% male, 45.2% female). None of the participants were smokers. Most participants were expatriates (84.5%) and lived in urban areas (56%) (Table 1).

**Table 1:** Sociodemographic characteristics of study participants.

Parameter	Study population n, 84 No. (%)
Age years	21.02 ± 1.31
Sex	
Male	46 (54.8%)
Female	38 (45.2%)
Smoking	
Smoker	0 (0%)
Non-smoker	84 (100%)
Expatriate	
Expatriate	71 (84.5%)
Non-Expatriate	13 (15.5%)
Residency	
Rural	37 (44%)
Urban	47 (56%)

Data are expressed as n (%).

The mean weight of the participants was 68.00 ± 11.76 kg, ranging from 44.10 to 97.60 kg. 2.4% of participants were underweight (< 50 kg), 60.7% had a normal weight (50–70 kg), 31.0% were overweight (70–90 kg), and 6.0% were obese (> 90 kg). This distribution highlights a substantial prevalence of excess weight, which is associated with metabolic and liver-related risks. The mean height was 169.21 ± 9.56 cm, ranging from 149.00 to 190.00 cm. 14.3% of participants were classified as short (< 160 cm), 41.7% as average (160–170 cm), and 44.0% as tall (> 170 cm). The height distribution is relevant for BMI calculations and body composition assessments. The mean BMI was 23.71 ± 3.87 kg/m^2^, ranging from 16.30 to 36.70 kg/m^2^. 4.8% of participants were underweight (BMI < 18.5), 60.7% had an optimum BMI (18.5–24.9), 27.4% were overweight (25–29.9), and 7.1% were obese (30 ≥). A significant proportion of participants are in the overweight and obese categories, suggesting potential risks for liver fat accumulation (Table 2).

**Table 2:** Anthropometric characteristics of study participants.

Parameter	Study population *n, 84* mean ± SD (Max–Min) No. (%)
Weight kg	68.00 ± 11.76 (44.10—97.60)
Underweight: < 50 kg	2 (02.4%)
Normal: 50–70 kg	51 (60.7%)
Overweight: 70–90 kg	26 (31.0%)
Obese: > 90 kg	5 (06.0%)
Height cm	169.21 ± 9.56 (149.00 – 190.00)
Short: < 160 cm	12 (14.3%)
Average: 160–170 cm	35 (41.7%)
Tall: > 170 cm	37 (44.0%)
Waist circumference cm	79.49 ± 8.78 (53.00 -102.00)
Female < 88 cm	34 (40.5%)
Female ≥ 88 cm	4 (04.8%)
Male < 102 cm	45 (53.6%)
Male ≥ 102 cm	1 (01.1%)
BMI kg/m2	23 0.71 ± 3.87 (16.30—36.70)
Underweight: < 18.5	4 (04.8%)
Optimum: 18.5 -24.9	51 (60.7%)
Overweight: 25 – 29.9	23 (27.4%)
Obese: ≥ 30	6 (07.1%)
BMR kcal/day	1447.7 ± 202.1 (1104—1868)
Female < 1381	33 (39.3%)
Female > 1381	5 (06.0%)
Male < 1744	38 (45.2%)
Male > 1744	8 (09.5%)
Muscle mass %	32.13 ± 5.64 (23.9 – 50.5)
Female < 31%	21 (25.0%)
Female 31–33%	12 (14.3%)
Female > 33%	5 (6.0%)
Male < 40%	39 (46.4%)
Male 40–44%	7 (08.3%)
Fat mass %	25.1 ± 8.91 (6.7—40.9)
Female < 18%	2 (02.4%)
Female 18–28%	2 (02.4%)
Female ≥ 29%	34 (40.5%)
Male < 10%	15 (17.9%)
Male 10–20%	7 (08.3%)
Male ≥ 21%	24 (28.6%)
Previous weight loss	
No	51 (60.7%)
Yes	33 (39.3%)
Weight loss in kg	
No	51 (60.7%)
< 5 kg loss	9 (10.7%)
≥ 5- < 10 kg loss	14 (16.7%)
≥ 10 kg loss	10 (11.9%)
Weight loss duration (W)	
No	51 (60.7%)
3–6 weeks	16 (19.0%)
7–10 weeks	7 (08.3%)
> 10 weeks	10 (11.9%)
Weight loss methods	
No weight loss	51 (60.7%)
Diet	19 (22.6%)
Medication (Glucophage)	2 (2.4%)
Sports	9 (10.7%)
All of them	3 (3.6%)
Weight gain recently	
No	58 (69%)
Yes	26 (31%)

Data are expressed as mean ± standard deviation (SD) and n (%).

The mean waist circumference was 79.49 ± 8.78 cm, ranging from 53.00 to 102.00 cm. 40.5% of females had a waist circumference below 88 cm, while 4.8% exceeded this threshold. Among males, 53.6% had a waist circumference below 102 cm, while 1.1% exceeded it. These values reflect a relatively low prevalence of central obesity. The mean BMR was 1447.7 ± 202.1 kcal/day, ranging from 1104 to 1868 kcal/day. 39.3% and 45.2% of participants had a BMR below sex-specific thresholds for females and males respectively, while 6% and 09.5% exceeded them. This reflects variations in resting energy expenditure, influenced by body composition and muscle mass **(**Table 2**)**.

The mean muscle mass percentage was 32.13 ± 5.64%, ranging from 23.9 to 50.5%. Among females, 25.0% had low muscle mass (sarcopenia: < 31%), 14.3% were within the normal range (31–33%), and 6.0% exceeded (muscle hypertrophy > 33%). Among males, 46.4% had low muscle mass (< 40%), while 8.3% were within the 40–44% range. These findings indicate a high prevalence of low muscle mass, particularly in males, which may contribute to metabolic dysfunction. The mean fat mass percentage was 25.1 ± 8.91%, ranging from 6.7 to 40.9%. Among females, 40.5% exceeded the healthy threshold (≥ 29%). Among males, 28.6% exceeded the threshold (≥ 21%). Elevated fat mass is a significant risk factor for both steatosis and metabolic-associated fatty liver disease (MAFLD) **(**Table 2**)**. More details on the socio-demographic data of the study population are found in Table 2.

### Clinical characteristics of the study population

Our study showed that 97.6% of participants had no chronic diseases. Only 1.2% had bronchial asthma, and 1.2% had Type 1 Diabetes Mellitus. Additionally, 63.1% of participants reported no history of Coronavirus infection, and 89.3% did not use continuous medications, reflecting the relatively young age of the study population (Table 3).

**Table 3:** Clinical characteristics of the study population.

Parameter	Study population *n, 84* No. (%)
Chronic Diseases	
No	82 (97.6%)
Bronchial asthma	1 (1.2%)
Diabetes Mellitus -1	1(1.2%)
Previous Corona infection	
No	53 (63.1%)
Yes	31 (36.9%)
Continuous medicine takes	
Absent	75(89.3%)
Vitamins	3 (3.6%)
Anti-acne	2 (2.4%)
Anti-histaminic	2 (2.4%)
Bronchodilators	1 (1.2%)
Multiple	1 (1.2%)
Family history of obesity	
No	51 (60.7%)
Yes	33 (39.3%)
Family history of chronic disease	
No	35 (41.7%)
Diabetes Mellitus (DM)	16 (19.0%)
Hypertension (HTN)	14 (16.7%)
DM & HTN	13 (15.5%)
DM, HTN & MAFLD	3 (3.6%)
DM & MAFLD	1 (1.2%)
Bronchial Asthma	1 (1.2%)
DM, HTN, & CHD	1 (1.2%)
Steatosis (CAP score: dB/m)	
Absent (S0: < 238)	63 (75%)
Mild (S1: 238 -260)	8 (9.5%)
Moderate (S2: 261–290)	10 (11.9%)
Severe (S3: > 290)	3 (3.6%)
Fibrosis (LMS: kPa)	
No fibrosis (F0 < 6.2)	60 (71.4%)
Mild fibrosis (F1 ≥ 6.2—7.6)	18 (21.4%)
Moderate fibrosis (F2 ≥ 7.6–8.8)	3 (3.6%)
Severe fibrosis (F3 ≥ 8.8–11.8)	3 (3.6%)
Cirrhosis (F4 ≥ 11.8)	0 (0%)

Data are n (%).

The study also showed that 39.3% of participants had a family history of obesity, while 60.7% did not. A significant proportion (58.3%) of participants reported a family history of chronic diseases, including diabetes, hypertension, coronary heart disease, and obesity, highlighting a potential genetic or environmental predisposition to metabolic and liver-related diseases within the cohort (Table 3).

Regarding grading and staging of steatosis and fibrosis, 75% of participants had no evidence of steatosis (S0), while 9.5% had mild steatosis (S1), 11.9% had moderate steatosis (S2), and 3.6% had severe steatosis (S3). Additionally, fibrosis was absent in 71.4% (F0), while 21.4% had mild fibrosis (F1), 3.6% had moderate fibrosis (F2), and 3.6% had severe fibrosis (F3). No cases of cirrhosis were observed (Table 3).

Based on the analysis, 63 students (75%) had CAP values < 238 dB/m (no steatosis), while 21 students (25%) had CAP values ≥ 238 dB/m (steatosis). Furthermore, 73 students (86.9%) did not have MAFLD, while 11 students (13.1%) were diagnosed with MAFLD, defined as steatosis (CAP ≥ 238 dB/m) plus metabolic dysfunction (WC > 102 cm in men, > 88 cm in women, or BMI > 25 kg/m^2^). The chi-square (X^2^) test showed a highly significant association between MAFLD and steatosis (*p* < 0.001). The Pearson correlation coefficient (r = 1, *p* < 0.001) indicated a strong positive correlation between MAFLD and steatosis frequency. Relative risk (RR) analysis showed that MAFLD occurs in approximately 52% of individuals with steatosis (OR = 2.1, 95% CI: 1.34–3.23), meaning that individuals with steatosis are twice as likely to develop MAFLD compared to those without steatosis **(**Table 3**)**.

### Lifestyle of the study population

Most participants (58.3%) reported being physically inactive, while only 41.7% engaged in regular physical activity. Unhealthy dietary patterns were reported by 61.9% of participants, characterized by frequent consumption of processed foods, sugary drinks, and high-fat meals. Only 38.1% reported adhering to a healthy diet that includes fresh fruits, vegetables, whole grains, and lean proteins. Poor sleep quality was reported by 46.4% of participants, while 53.6% reported good sleep quality. The lifestyle characteristics of the study population reveal a high prevalence of physical inactivity, unhealthy dietary habits, and poor sleep quality, all of which are significant contributors to metabolic dysfunction and liver disease (Table 4).

**Table 4:** Lifestyle characteristics of the study population.

Parameter	`Study population *n, 84* No. (%)
Sleeping habits	
Poor sleep	39 (46.4%)
Healthy sleep	45 (53.6%)
Exercise	
Inactive (sedentary)	49 (58.3%)
Active (regular)	35 (41.7%)
Dietary habits	
Unhealthy foods	52 (61.9%)
Healthy foods	32 (38.1%)

Data are n (%).

### The study population characteristics in relation to steatosis and MAFLD

**Fig. 2:**
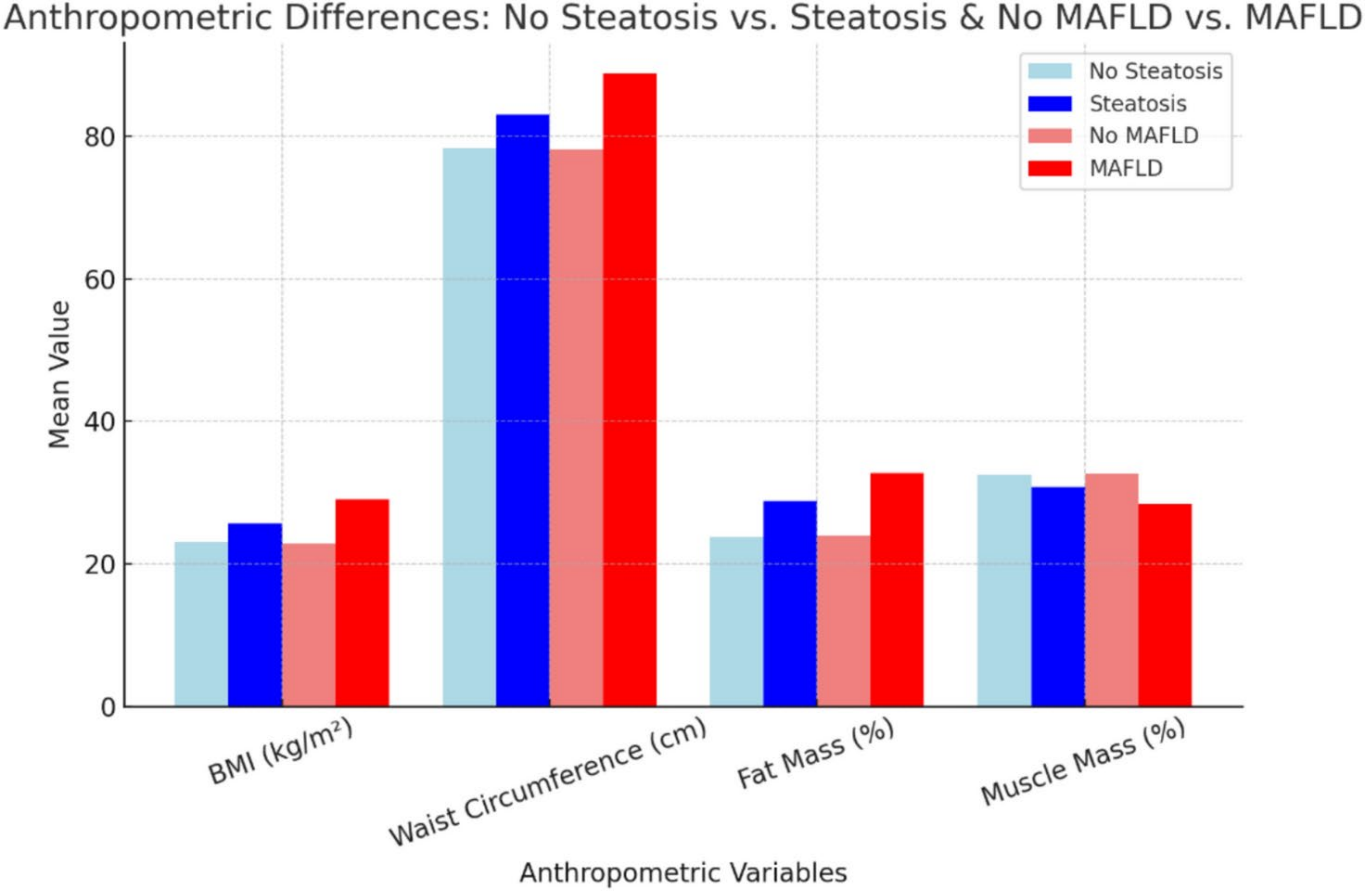
Anthropometric Differences in Steatosis and MAFLD Groups.

**Fig. 3:**
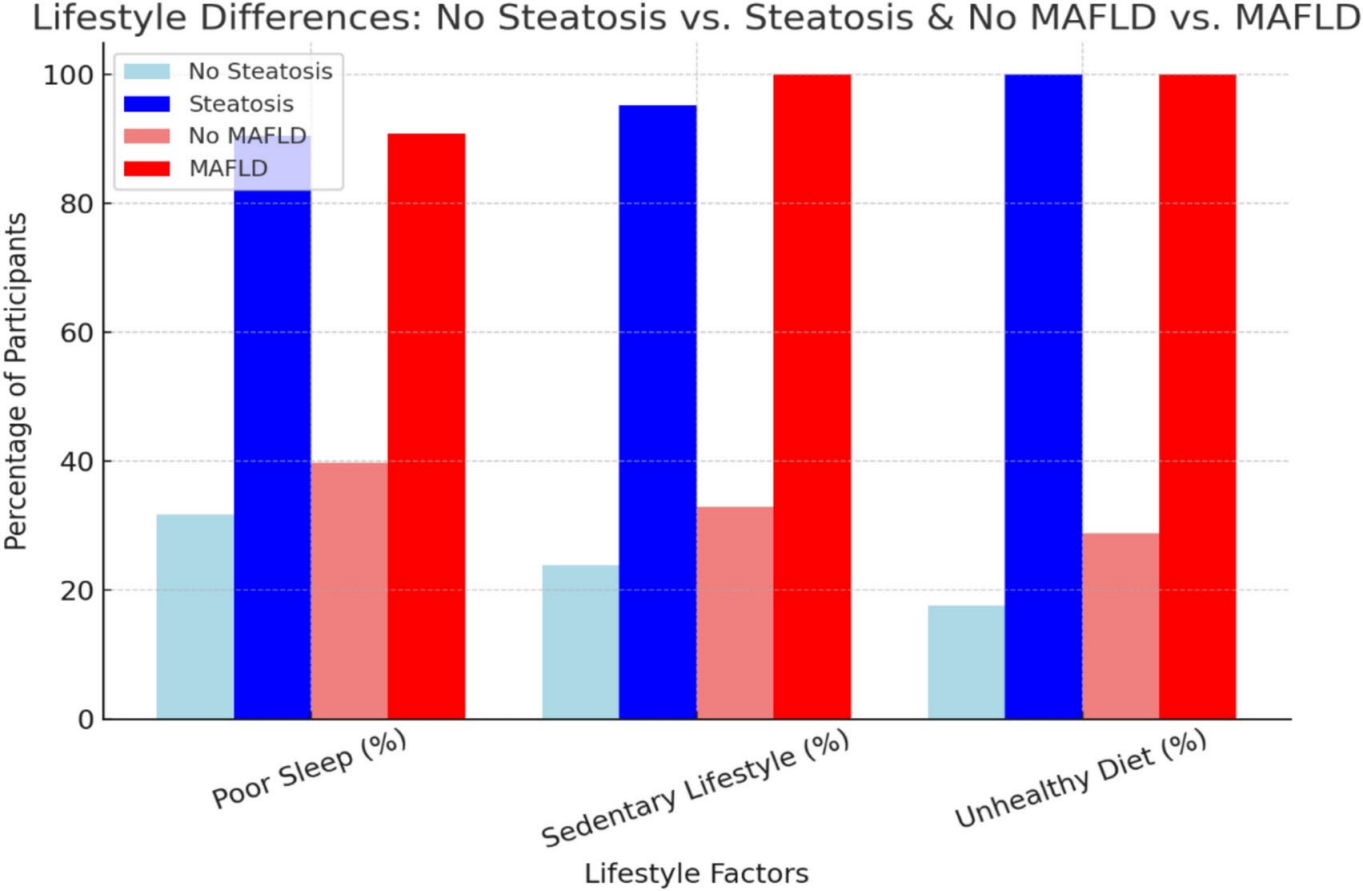
Lifestyle Factors and Their Association with Steatosis and MAFLD.

Participants with steatosis exhibited significantly higher BMI (25.67 ± 4.58 vs. 23.05 ± 3.40 kg/m^2^, *p* < 0.001), weight (73.67 ± 13.37 vs. 66.11 ± 10.92 kg, *p* = 0.01), waist circumference (83.05 ± 9.57 vs. 78.30 ± 8.25 cm, p = 0.03), fat mass percentage (28.83 ± 8.09% vs. 23.81 ± 8.88%, *p* = 0.02), and BMR (1494.43 ± 215.08 vs. 1432.13 ± 96.8 kcal/day, *p* = 0.03) compared to those without steatosis. Additionally, participants with steatosis reported more frequent weight regain (47.6% vs. 25.4%, *p* = 0.05). These findings highlight a strong association between steatosis and obesity-related metrics **(**Table 5**, **Fig. 2**)**. Participants with steatosis had a significantly higher prevalence of a family history of obesity (57.1%) compared to those without steatosis (33.3%, *p* = 0.05) **(**Table 6**).** Significant differences in lifestyle patterns, including sleeping, exercise, and dietary habits, were observed between participants with and without steatosis. Poor sleep was reported by 90.5% of participants with steatosis compared to 31.7% in the no steatosis group (p = 0.002). A sedentary lifestyle was found in 95.2% of participants with steatosis, compared to 23.8% of those without steatosis (p < 0.001). Additionally, 100% of participants with steatosis reported unhealthy dietary habits, while 17.5% of those without steatosis followed an unhealthy diet (*p* < 0.001) **(**Table 7**, **Fig. 3**)**.

**Table 5:** Demographic and anthropometric characteristics of the study population with/without steatosis and MAFLD.

Variables	No steatosis (n = 63; 75%)	Steatosis (n = 21; 25%)	*P* value	No MAFLD (n = 73; 87%)	MAFLD (n = 11; 13%)	*P* value
Age	21.03 ± 1.33	21.00 ± 1.26	0.92	21.05 ± 1.29	20.82 ± 1.47	0.58
Sex			0.80			0.99
Male	35 (55.6%)	11(52.4%)		40 (54.8%)	6 (54.5%)	
Female	28 (24.4%)	10 (47.6%)		33 (45.2%)	5 (45.5%)	
Expatriate			0.60			0.54
Expatriate	54 (85.7%)	17 (81%)		62 (84.9%)	9 (81.8%)	
Non-Expatriate	9 (14.3%)	4 19.0%)		11 (15.1%)	2 (18.2%)	
Residency			0.80			0.59
Rural	27 (42.9%)	10 (47.6%)		32 (43.8%)	5 (45.5%)	
Urban	36 (57.1%)	11 (52.4%)		41 (56.2%)	6 (54.5%)	
Weight kg	66.11 ± 10.92	73.67 ± 13.37	**0.01**	65.84 ± 10.61	82.34 ± 10.78	**0.000**
Height cm	169.16 ± 9.74	169.38 ± 9.26	0.93	169.33 ± 9.45	168.45 ± 10.75	0.78
BMI kg/m2	23.05 ± 3.40	25.671 ± 4.58	**0.00**	22.90 ± 3.23	29.05 ± 3.66	**0.000**
WC	78.30 ± 8.25	83.05 ± 9.57	**0.03**	78.10 ± 7.96	88.73 ± 8.73	**0.000**
BMR kcal/day	1432.13 ± 96.8	1494.43 ± 215.08	**0.01**	1429.86 ± 93.44	1566.09 ± 27.37	**0.03**
Muscle mass %	32.52 ± 5.61	30.80 ± 5.94	0.22	32.65 ± 5.53	28.37 ± 5.73	**0.02**
Fat mass %	23.81 ± 8.88	28.83 ± 8.09	**0.02**	23.91 ± 8.60	32.74 ± 7.25	**0.00**
Past weight loss			0.37			0.07
No	40 (63.5%)	11 (52.4%)		47 (64.4%)	4 (36.4%)	
Yes	23 (36.5%)	10 (47.6%)		26 (35.6%)	7 (63.6%)	
Weight loss kg	3.37 ± 7.78	4.19 ± 5.68	0.66	3.11 ± 7.32	6.64 ± 6.61	0.14
WL method			0.83			0.07
No loss	40 (63.5%)	11 (52.4%)		48 (65.8%)	3 (27.3%)	
Diet	14 (22.2%)	5 (23.8%)		14 (19.2%)	5 (45.5%)	
Glucophage	1 (1.6%)	1 (4.8%)		1 (1.4%)	1 (9.1%)	
Sports	6 (9.5%)	3 (14.3%)		8 (11.0%)	1 (9.1%)	
All of them	2 (3.2%)	1 (4.8%)		2 (2.7%)	1 (9.1%)	
WL duration			0.13			**0.02**
No loss	41 (65.1%)	11 (52.4%)		48 (65.8%)	3 (27.3%)	
3–6 w	9 (14.3%)	7 (33.3%)		11 (15.1%)	5 (45.5%)	
7–10 w	6 (9.5%)	0 (0.0%)		6 (8.2%)	0 (0.0%)	
> 10 w	7 (11.1%)	3 (14.3%)		8 (11.0%)	3 (27.3%)	
Weight re-gain			**0.05**			**0.01**
No	47 (74.6%)	11 (52.4%)		54 (74.0%)	4 (36.4%)	
Yes	16 (25.4%)	10 (47.6%)		19(26.0%)	7 (63.6%)	

Abbreviations: BMI, body mass index; WC, Waist circumference; BMR, Basal metabolic rate; WL, Weight loss. Data are presented as n (%) for categorical variables and mean ± SD for normally distributed continuous variables (assessed by Shapiro–Wilk test). Independent t-test was used to compare continuous variables. Pearson’s Chi-Square test was used for categorical variables, with Fisher’s Exact Test applied for expected counts < 5.

**Table 6:** Clinical characteristics of the study population with/without steatosis and MAFLD.

Variables	No steatosis (n = 63; 75%)	Steatosis (n = 21; 25%)	*P* value	No MAFLD (n = 73; 87%)	MAFLD (n = 11; 13%)	*P* value
Previous CoV-2			0.67			0.48
No	39 (61.9%)	14 (66.7%)		45 (61.6%)	8 (72.7%)	
Yes	24 (38.1%)	7 (33.3%)		28 (38.4%)	3 (27.3%)	
Medications			0.82			0.91
Absent	55 (87.3%)	20 (95.2%)		64 (87.7%)	11 (100%)	
Vitamins	2 (3.2%)	1 (4.8%)		2 (2.7%)	0 (0.0%)	
Anti-acne	2 (3.2%)	0		3 (4.1%)	0 (0.0%)	
Antihistamines	2 (3.2%)	0		1 (1.4%)	0 (0.0%)	
Bronchodilators	1 (1.6%)	0		2 (2.7%)	0 (0.0%)	
Multiple	1 (1.6%)	0		1 (1.4%)	0 (0.0%)	
Family obesity			**0.05**			**0.05**
No	42 (66.7%)	9 (42.9%)		47 (64.4%)	4 (36.4%)	
Yes	21 (33.3%)	12 (57.1%)		26 (35.6%)	7 (63.6%)	
Family comorbidities			0.57			0.85
No	26 (41.3%)	9 (42.9%)		31 (42.5%)	4 (36.4%)	
DM	9 (14.3%)	7 (33.3%)		12 (16.4%)	4 (36.4%)	
HTN	11 (17.5%)	3 (14.3%)		12 (16.4%)	2 (18.2%)	
DM, HTN	11 (17.5%)	2 (9.5%)		12 (16.4%)	1 (9.1%)	
DM, HTN, MAFLD	3 (4.8%)	0 (0.0%)		3 (4.1%)	0 (0.0%)	
DM, MAFLD	1 (1.6%)	0 (0.0%)		1 (1.4%)	0 (0.0%)	
BA	1 (1.6%)	0 (0.0%)		1 (1.4%)	0 (0.0%)	
DM, HTN, CHD	1 (1.6%)	0 (0.0%)		1 (1.4%)	0 (0.0%)	

Abbreviations: CoV-2, severe acute respiratory syndrome coronavirus 2 (SARS-CoV-2); HTN, Hypertension; MAFLD, Metabolic dysfunction-associated fatty liver disease; CHD, Coronary heart disease. Data are presented as n (%) for categorical variables and mean ± SD for normally distributed continuous variables (assessed by Shapiro–Wilk test). Independent t-test was used to compare continuous variables. Pearson’s Chi-Square test was used for categorical variables, with Fisher’s Exact Test applied for expected counts < 5.

**Table 7:** Lifestyle of the study population with/without steatosis and MAFLD.

Variables	No steatosis (n = 63; 75%)	Steatosis (n = 21; 25%)	*P* value	No MAFLD (n = 73; 87%)	MAFLD (n = 11; 13%)	*P* value
Sleeping habits			**0.000**			**0.002**
Poor sleep	20 (31.7%)	19 (90.5%)		29 (39.7%)	10 (90.9%)	
Good sleep	43 (68.3%)	2 (9.5%)		44 (60.3%)	1 (9.1%)	
Exercise			**0.000**			**0.000**
Sedentary	15 (23.8%)	20 (95.2%)		24 (32.9%)	11 (100%)	
Active	48 (76.2%)	1 (4.8%)		49 (67.1%)	0 (0.0%)	
Dietary habits			**0.000**			**0.000**
Unhealthy	11 (17.5%)	21 (100%)		21 (28.8%)	11 (100%)	
Healthy	52 (82.5%)	0 (0.0%)		52 (71.2%)	0 (0.0%)	

Data are n (%), Pearson’s Chi-Square test for categorized variables. Note: Dietary habits may be subject to spectrum bias, as participants with MAFLD may have altered their diet prior to the study

Participants with MAFLD exhibited significantly higher BMI (29.05 ± 3.66 vs. 22.90 ± 3.23 kg/m^2^, *p* < 0.001), weight (82.34 ± 10.78 vs. 65.84 ± 10.61 kg, *p* < 0.001), waist circumference (88.73 ± 8.73 vs. 78.10 ± 7.96 cm, *p* < 0.001), fat mass percentage (32.74 ± 7.25% vs. 23.91 ± 8.60%, *p* < 0.001), and BMR (1566.09 ± 27.37 vs. 1429.86 ± 93.44 kcal/day, p < 0.001) compared to those without MAFLD. A significant reduction in muscle mass percentage (28.37 ± 5.73% vs. 32.65 ± 5.53%, *p* = 0.02) was also observed in MAFLD, indicating sarcopenic obesity as a contributing factor. The duration of weight loss was significantly longer in MAFLD participants (*p* = 0.02), and the prevalence of weight regain was significantly higher (63.6% vs. 26%, *p* = 0.01) in MAFLD patients compared to those without MAFLD, highlighting the difficulty in maintaining weight loss in these individuals (Table 5).

Participants with MAFLD had a significantly higher prevalence of a family history of obesity (63.6%) compared to those without MAFLD (35.6%, *p* = 0.05) (Table 6). Lifestyle factors also differed significantly between groups. Poor sleep was reported by 90.9% of participants with MAFLD compared to 39.7% of those without MAFLD (*p* = 0.002). All participants (100%) with MAFLD had a sedentary, inactive lifestyle, compared to 32.9% in those without MAFLD (*p* < 0.001). Similarly, all participants (100%) with MAFLD followed unhealthy dietary habits, while 28.8% of those without MAFLD had an unhealthy diet (*p* < 0.001) (Table 7).

### The study population characteristics across steatosis grades and fibrosis stages

Key variables associated with steatosis severity and fibrosis stages were analyzed to assess their impact on disease progression. A significant increase in BMI (p = 0.01), waist circumference (p = 0.01), and fat mass percentage (p = 0.01) was observed across steatosis severity grades (S1–S3). Participants with severe steatosis (S3) exhibited the highest values, suggesting a progressive link between obesity and hepatic fat accumulation. Additionally, lifestyle factors—including poor sleep, physical inactivity, and unhealthy dietary habits—were prevalent across all grades of steatosis. A strong association was observed between these factors and advanced steatosis severity (S3: 100% for each factor, p < 0.001) (Table 8).

**Table 8:** Key Variables characteristics of the study population across steatosis grades.

	S0 (n = 63; 75%)	S1 (n = 8; 9.5%)	S2 (n = 10; 11.9%)	S3 (n = 3; 3.6%)	*P* value
Weight Kg	66.11 ± 10.92	71.81 ± 14.12	75.42 ± 15.11	72.77 ± 5.65	0.08
BMI kg/m2	23.05 ± 3.40	24.28 ±	25.72 ± 5.52	29.20 ± 3.70	**0.01**
WC cm	78.30 ± 8.25	82.13 ± 10.84	84.20 ± 10.05	88.11 ± 3.53	**0.01**
Fat mass %	23.81 ± 8.88	28.86 ± 5.36	25.78 ± 8.83	38.93 ± 1.70	**0.01**
Weight re-gain					0.26
No	47 (74.6%)	4 (50.0%)	5 (50.0%)	2 (66.7%)	
Yes	16 (25.4%)	4 (50.0%)	5 (50.0%)	1 (33.3%)	
Family obesity					0.25
No	42 (66.7%)	4 (50.0%)	4 (40.0%)	1 (33.3%)	
Yes	21 (33.3%)	4 (50.0%)	6 (60.0%)	2 (66.7%)	
Sleeping habits					**0.000**
Poor sleep	20 (31.7%)	7 (87.5%)	9 (90.0%)	3 (100.0%)	
Healthy sleep	43 (68.3%)	1 (12.5%)	1 (10.0%)	0 (0.0%)	
Exercise					**0.000**
Sedentary	15 (23.8%)	7 (87.5%)	10 (100%)	3 (100%)	
Active	48 (76.2%)	1 (12.5%)	0 (0.0%)	0 (0.0%)	
Dietary habits					**0.000**
Unhealthy	11 (17.5%)	8 (100%)	10 (100%)	3 (100%)	
Healthy	52 (82.5%)	0 (0.0%)	0 (0.0%)	0 (0.0%)	

Abbreviations: BMI: Body Mass Index, WC: Waist Circumference, Data are n (%), mean ± SD for normally distributed data as determined by the Shapiro–Wilk test. *Pearson’s Chi-Square test for categorized variables. One-way analysis of variance (ANOVA) for continuous variables.

For fibrosis progression, poor sleep, physical inactivity, and unhealthy dietary habits were significantly more common in participants with advanced. These findings underscore the crucial role of lifestyle factors in fibrosis development (Table 9).

**Table 9:** Key Variables characteristics of the study population across fibrosis stages.

	F0 (n = 60; 71.4%)	F1 (n = 18; 21.4%)	F2 (n = 3; 3.6%)	F3 (n = 3; 3.6%)	F4 (n = 0; 0.0%)	*P* value
Weight Kg	67.89 ± 12.95	67.77 ± 8.43	60.80 ± 7.21	78.70 ± 8.83	-	0.33
BMI kg/m2	23.85 ± 4.25	23.33 ± 3.07	22.03 ± 0.961	24.87 ± 0.87	-	0.79
WC cm	79.62 ± 9.45	80.06 ± 7.14	71.33 ± 3.79	81.67 ± 2.89	-	0.42
BMR kcal/day	1437.28 ± 203.4	1458.50 ± 184.2	1403.67 ± 227.8	1625.33 ± 269.0	-	0.40
Muscle mass %	31.55 ± 5.18	33.27 ± 6.99	33.30 ± 5.02	34.63 ± 9.18	-	0.57
Fat mass %	25.60 ± 8.68	24.13 ± 9.67	20.05 ± 11.78	25.00 ± 9.29	-	0.72
WL duration						0.43
No loss	36 (60.0%)	12 (66.7%)	1 (33.3%)	2 (66.7%)	-	
3–6 w	10 (16.7%)	5 (27.8%)	1 (33.3%)	0 (0.0%)	-	
7–10 w	5 (8.3%)	0 (0.0%)	1 (33.3%)	0 (0.0%)	-	
> 10 w	9 (15.0%)	1 (5.6%)	0 (0.0%)	1 (33.3%)	-	
Weight re-gain						0.85
No	40 (66.7%)	14 (77.8%)	2 (66.7%)	2 (66.7%)	-	
Yes	20 (33.3%)	4 (22.2%)	1 (33.3%)	1 (33.3%)	-	
Chronic Diseases						0.99
No	58 (96.7%)	18 (100.0%)	3 (100.0%)	3 (100.0%)	-	
BA	1 (1.7%)	0 (0.0%)	0 (0.0%)	0 (0.0%)	-	
DM-1	1 (1.7%)	0 (0.0%)	0 (0.0%)	0 (0.0%)	-	
Family obesity						0.30
No	40 (66.7%)	9 (50.0%)	1 (33.3%)	1 (33.3%)	-	
Yes	20 (33.3%)	9 (50.0%)	2 (66.7%)	2 (66.7%)	-	
Sleeping habits						**0.05**
Poor sleep	26 (43.3%)	10 (55.6%)	1 (33.3%)	2 (66.7%)	-	
Healthy sleep	34 (56.7%)	8 (44.4%)	2 (66.7%)	1 (33.3%)	-	
Exercise						
Sedentary	22 (36.7%)	10 (55.6%)	1 (33.3%)	2 (66.7%)	-	**0.04**
Active	38 (63.3%)	8 (44.4%)	2 (66.7%)	1 (33.3%)	-	
Dietary habits						
Unhealthy	20 (33.3%)	9 (50.0%)	0 (0.0%)	3 (100.0%)	-	**0.03**
Healthy	40 (66.7%)	9 (50.0%)	3 (100.0%)	0 (0.0%)	-	

Abbreviations: BMI, Body mass index; WC, waist circumference; BMR, Basal metabolic rate; WL, Weight loss; BA, Bronchial Asthma; DM-1, Diabetes Mellitus type 1. Data are n (%), mean ± SD for normally distributed data as determined by the Shapiro–Wilk test. Pearson’s Chi-Square test for categorized variables. One-way analysis of variance (ANOVA) for continuous variables.

The regression analysis provided insights into the significant risk factors for both steatosis and MAFLD. Weight, BMI, waist circumference, and fat mass percentage were positively associated with both conditions, indicating that higher body weight, BMI, WC, and fat mass % significantly increase the odds of developing steatosis and MAFLD. The odds ratios (ORs) were notably higher for MAFLD compared to steatosis, reflecting a stronger association with MAFLD (OR = 1.14, *p* = 0.01; OR = 1.63, *p* = 0.01; OR = 1.15, *p* = 0.03; OR = 1.16, *p* = 0.01, respectively) (Table 10).

**Table 10:** Binary logistic regression analysis for the parameters affecting steatosis and MAFLD.

	Steatosis			MAFLD		
	*P* value	OR	95% C. I	*P* value	OR	95% C. I
Weight kg	**0.01**	1.05	1.01–1.10	**0.000**	1.14	1.06–1.22
BMI kg/m2	**0.01**	1.19	1.04–1.36	**0.000**	1.63	1.25–2.11
WC cm	**0.03**	1.06	1.00–1.12	**0.001**	1.15	1.06–1.26
BMR kcal/day	**0.01**	1.19	1.04–1.36	**0.04**	1.00	1.00–1.07
Muscle mass %	0.24	0.94	0.85–1.04	**0.02**	0.79	0.66–0.96
Fat mass %	**0.02**	1.07	1.00–1.14	**0.00**	1.16	1.04–1.29
WL duration						
No (reference)						
3–6 w	0.54	1.62	0.14–2.82	**0.03**	1.14	0.02–0.85
7–10 w	0.48	1.82	0.34–9.69	0.95	1.06	0.19–5.90
> 10 w				0.99		
Family obesity						
No (reference)						
Yes	**0.05**	2.67	0.97–7.33	0.08	1.32	0.08–1.12
Sleeping habits						
Poor sleep	**0.000**	2.43	4.33–6.29	**0.01**	5.17	1.98–10.7
Healthy (reference)						
Exercise						
Sedentary	**0.000**	7.69	3.06–22.3	**0.000**	1.17	1.08–1.28
Active (reference)						
Dietary habits						
Unhealthy	**0.000**	4.55	1.62–16.3	**0.000**	1.01	1.0–1.02
Healthy (reference)						

Abbreviations: BMI, Body mass index; WC, waist circumference; BMR, Basal metabolic rate; WL, Weight loss; OR, Odd`s ratio; C.I, Confidence interval. Statistically significant at *p* ≤ 0.05.

Muscle mass percentage was significantly associated with MAFLD (OR = 0.79, *p* = 0.02) but not with steatosis. This inverse relationship suggests that higher muscle mass reduces MAFLD risk by 21%, emphasizing the importance of strategies aimed at increasing or maintaining muscle mass (Table 10).

A sedentary lifestyle was a strong predictor for both conditions (OR = 7.69, *p* = 0.000 for steatosis; OR = 1.17, *p* = 0.01 for MAFLD). Similarly, unhealthy dietary practices were associated with increased risk (OR = 4.55, p = 0.000 for steatosis; OR = 1.01, p = 0.01 for MAFLD). Poor sleep significantly increased the risk for steatosis (OR = 2.43, *p* = 0.000) and MAFLD (OR = 5.17, *p* = 0.01) (Table 10).

Short weight-loss durations (3–6 weeks) significantly increased MAFLD risk (OR = 1.14, *p* = 0.03), but no significant effect was observed for steatosis. The family history of obesity showed borderline significance for steatosis (p = 0.05) but was not significant for MAFLD (Table 10).

**Fig. 4:**
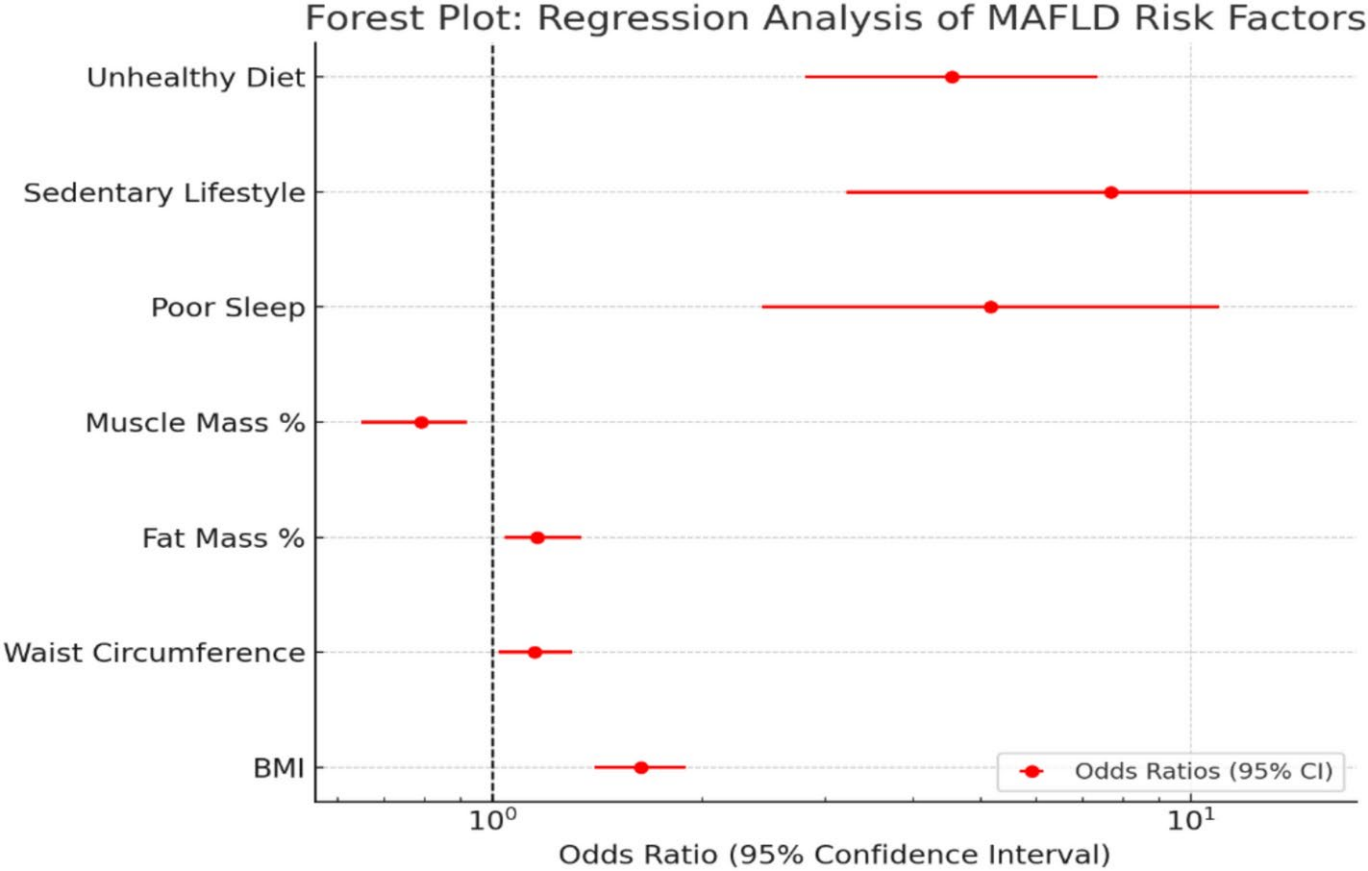
Forest plot showing odds ratios (OR) with 95% confidence intervals (CI) for key predictors of MAFLD and steatosis, including BMI, waist circumference, fat mass percentage, muscle mass percentage, poor sleep, sedentary lifestyle, and unhealthy diet.

The findings highlight that modifiable risk factors influence both conditions, but MAFLD exhibits stronger associations with metabolic parameters and lifestyle factors. Efforts targeting weight management, physical activity, dietary improvements, and better sleep habits are essential in reducing risks for steatosis and MAFLD. Forest plot displaying the odds ratios (OR) with 95% confidence intervals (CI) for key predictors of MAFLD and steatosis. The predictors include BMI, waist circumference, fat mass percentage, muscle mass percentage, poor sleep, sedentary lifestyle, and unhealthy diet. OR values greater than 1 indicate an increased risk, while OR values less than 1 suggest a protective effect. The dashed vertical line at OR = 1 represents no association. A log scale is used for better visualization of effect sizes **(**Fig. 4**)**.

## Discussion

In 2020, a new term, metabolic-associated fatty liver disease (MAFLD), was coined to better describe fatty liver disease linked to metabolic abnormalities. It seeks to enhance the disease’s diagnostic standards and individualized treatment plans, replacing the antiquated term nonalcoholic fatty liver disease (NAFLD). Unlike NAFLD, MAFLD does not require the exclusion of other etiologies of liver disease, such as excessive alcohol consumption or viral hepatitis. MAFLD is diagnosed in patients when they have both “hepatic steatosis” and any of the following three metabolic conditions: “overweight/obesity”, “diabetes mellitus”, or evidence of “metabolic dysregulation in lean/normal weight individuals”^19^. The original definition was established in 1980 and has remained unchanged since then. However, NAFLD is the most common liver disease in Western industrialized nations, affecting about 1 billion people globally, and its prevalence has been rising gradually^32^. MAFLD includes a wide spectrum of liver injuries, including nonalcoholic fatty liver (NAFL) and nonalcoholic steatohepatitis (NASH), depending on the presence of liver inflammation, which is observed only in NASH. This is of particular concern since NASH can progress to cirrhosis and hepatocellular carcinoma (HCC) and ultimately result in mortality, placing a significant burden on individuals, families, societies, and healthcare systems^13^.

Even though a liver biopsy is considered the golden method for diagnosing MAFLD and liver fibrosis, it cannot be used for mass screening because of its invasive nature and associated complications^33^. Rather, transient elastography (TE) has gained recognition as a valid, safe, and effective method for the evaluation of hepatic fat and fibrosis^34^. TE showed the ability to quantify liver stiffness and controlled attenuation parameter (CAP) scores, which correlate with fibrosis and hepatic fat content, respectively^33^. It also demonstrated high sensitivity and specificity for diagnosing hepatic steatosis and fibrosis early, emphasizing the role of TE in identifying moderate-to-severe steatosis in resource-limited settings, as a cost-effective alternative to liver biopsy in population-level screening in both research and clinical settings due to its non-invasive nature and ability to provide reliable quantitative measures^35^.

In this study, using transient elastography and bioimpedance analysis, MFLD cases are cases with hepatic steatosis (CAP ≥ 238 dB/m) plus WC > 102 in men and > 88 in females or overweight/obesity (BMI > 25), our work found in a group of young, seemingly healthy medical students, ages 18 to 22, that most participants (75%) showed no evidence of steatosis (S0), while 9.5% had mild steatosis (S1), 11.9% had moderate steatosis (S2), and 3.6% had severe steatosis (S3). In addition, Fibrosis was absent in 71.4% of participants (F0), while 21.4% had mild fibrosis (F1), 3.6% had moderate fibrosis (F2), 3.6% had severe fibrosis (F3), and no cases of cirrhosis were observed. A 13.1% frequency of metabolic-associated fatty liver disease (MAFLD). This provides comprehensive diagnostic insights. This represents a growing burden of MAFLD in younger populations, especially in middle-income nations where obesity and sedentary lifestyles are on the rise^36^, even though it is lower than global projections of 25–30%^13^.

We selected the age of 18 and above as the eligibility age for sample inclusion since various research have suggested that this is the age at which high onset NAFLD begins^37^. In the general population, the prevalence of fatty liver increases with age, rising from 1 to 3 percent in children^38^, 5 percent in adolescents^39^, 18 percent in people aged 20 to 40, 39 percent in people aged 40 to 50, and more than 40 percent in people aged 70 and older^40^. In addition to the greater prevalence rate of NAFLD in individuals over 40, the mortality rate was shown to be higher in people over 60^41^.

According to a study conducted on 19–21-year-old Egyptian university students using transient elastography (FibroScan®), 31.6% of them had steatosis, of which 57.9% had S3 (severe) steatosis, and 5% had fibrosis^42^. Another study indicated that 63.6% of Egyptian people, aged between 40 and 60, with metabolic disorders including hypertension or hyperlipidemia, had moderate-to-severe steatosis. Trans-abdominal ultrasound was used to grade the liver steatosis severity caused by MAFLD. The results showed that 36%, 34%, and 30% of the population had hepatic steatosis grades I (mild), II (moderate), and III (severe), respectively^20^. Furthermore, a different study conducted in Egypt using transient elastography discovered that obesity and metabolic dysfunction were the main causes of the 15.5% prevalence of MAFLD in non-obese people^43^. In line with the known age-related progression of MAFLD, nearly all earlier Egyptian investigations focused on older populations (≥ 40 years) and found greater prevalence rates. In line with international studies on metabolic and lifestyle factors, the results of such studies showed a robust correlation between MAFLD and increased BMI, waist circumference, and poor sleep quality.

In Africa, a systematic review estimated the prevalence of NAFLD at 13.5%, ranging from 9% in Nigeria to 20% in Sudan. Such studies primarily used ultrasonography or liver biopsy for diagnosis^44^. In a clinic-based South African study, NAFLD, simple steatosis, NASH, and severe liver fibrosis were found to be 87, 51, 36, and 17% prevalent, respectively, in a South African study. Participants ranged in age from 40 to 70 years, and the diagnostic criterion was liver biopsy^45^. There is currently not enough research describing the overall prevalence of MAFLD in the Middle East and North Africa (MENA) region. Nevertheless, the region’s high reported incidence of NAFLD (31.8%) points to a significant burden of MAFLD as well^46^. According to a retrospective study conducted in a Saudi Arabian tertiary care hospital, based on the radiology reports, the prevalence of MAFLD in individuals between the ages of 30 and 60 is close to 43%, highlighting the effects of high obesity rates and sedentary lifestyles^37^.

Globally, in a cohort study in the United States adolescent population aged from 13 to 17 years, Steatosis (S1-S3), based on CAP, and advanced fibrosis (F3-F4), based on TE, were present in 27% and 2.84%^47^**,** respectively. Studies in the United States, Europe, and Asia corroborate the trend, with significant MAFLD prevalence among non-obese individuals, underscoring the importance of metabolic dysfunction as a primary driver^48^. In the United States, the prevalence of MAFLD ranged from 25.9 to 39.1%^48–50^. South America is known to have a high prevalence of NAFLD (30.5%)^8^. As for Europe, a study that investigated a large cohort in the United Kingdom (423,252 individuals) found that 38.0% of the participants had MAFLD^51^. An extensive systematic review and meta-analysis involving over 13 million individuals in Asia reported a prevalence of 29.62% for MAFLD^9^.

Our study demonstrated that MAFLD has the potential to progress to metabolic dysfunction–associated steatohepatitis (MASH) and proceed to hepatic fibrosis. Pearson correlation coefficient (*r* = 1, *p* = 0.000) indicated a significant positive correlation between MAFLD cases and the prevalence of steatosis. The relative risk (RR) analysis indicated that MAFLD occurs in approximately 52% of cases with steatosis (OR = 2.1, 95% CI: 1.34, 3.23), meaning MAFLD is significantly two times more likely to happen in individuals with steatosis compared to individuals without steatosis. The progression of steatosis and fibrosis in the study was significantly associated with metabolic, anthropometric, and lifestyle factors. BMI was identified as one of the key factors with higher adiposity and particularly visceral fat deposition enhancing hepatic lipotoxicity and steatosis as well as fibrosis. Central obesity, in terms of waist circumference, had a similar effect since visceral fat directly affects hepatic fat by increasing the delivery of free fatty acids^52^. Sleep quality also plays a role since poor sleep quality affects the circadian rhythm and glucose metabolism and increases systemic inflammation, which in turn leads to hepatic steatosis and fibrosis. Also, physical inactivity and an unhealthy diet, including a high intake of refined carbohydrates and fats, were major factors that enhanced lipogenesis and fat deposition in the liver^53^. Sarcopenic obesity, where one has high-fat mass and low muscle mass percentages, was also another major factor as it is associated with increased insulin resistance and systemic inflammation, which fast tracks the disease. Surprisingly, weight loss was linked to fibrosis progression, and this could be because free fatty acids released during rapid weight loss induce inflammation of the liver^54^. Elevated levels of free fatty acids within the liver provide a source of oxidative stress by peroxisomal b-oxidation. As a result, hydrogen peroxide is produced. When iron is present, extremely reactive hydroxyl radicals are created. The release of these free radicals contributes to mitochondrial damage and the progression of liver fibrosis^55^. The results of our study demonstrate that MAFLD is a complex disease with many factors that influence the development and progression of steatosis and fibrosis, thus emphasizing the need for management of the modifiable risk factors, including BMI, waist circumference, physical inactivity, unhealthy diet, and sleep quality. Educating the public on the dangers of extreme weight loss techniques and encouraging the development of behavior change programs that consider these factors may help minimize the incidence of severe liver disease.

There are currently several methods and instruments for determining body composition^56^. The gold standard for determining body composition is currently dual-energy X-ray absorptiometry (DEXA), which offers precise measurements of lean body mass and regional body fat along with many benefits, such as high measurement reproducibility, observer independence, and little patient cooperation^57^. However, its viability for extensive population investigations is restricted by its high cost, intricate operational requirements, and logistical difficulties^58^. Compared to existing body composition instruments such as Dual-energy X-ray Absorptiometry (DEXA), the InBody 270 bioelectrical impedance analysis (BIA) device has several advantages. Unlike DEXA, it is non-invasive, portable, and radiation-free, which makes it safe for repeated usage and appropriate for vulnerable groups, including expectant mothers and children. Furthermore, it is inexpensive and requires little training, enabling speedy evaluations in a variety of contexts, such as public health screenings and clinics. InBody 270 offers accurate assessments of body fat percentage, skeletal muscle mass, and visceral fat—all of which are vital for evaluating obesity and metabolic health risks—while DEXA is more accurate for regional fat distribution and bone density. It is a useful option for tracking therapies and assessing changes in body composition because of its capacity to produce comprehensive reports and track longitudinal data. Although it is somewhat affected by hydration levels, its usability and accessibility make it a vital tool for clinical practice and research ^59^.

In our study, anthropometric measurements using InBody 270 revealed that participants with metabolic-associated fatty liver disease (MAFLD) demonstrated significantly higher anthropometric and metabolic values compared to their non-MAFLD counterparts. Body weight was higher in MAFLD participants (82.34 ± 10.78 kg vs. 65.84 ± 10.61 kg, *p* < 0.001), as was body mass index (BMI, 29.05 ± 3.66 kg/m^2^ vs. 22.90 ± 3.23 kg/m^2^, *p* < 0.001), reflecting a notable association with general obesity. Waist circumference “WC” (88.73 ± 8.73 cm vs. 78.10 ± 7.96 cm, *p* < 0.001) indicated increased central adiposity among MAFLD patients, while fat mass percentage was significantly elevated (32.74 ± 7.25% vs. 23.91 ± 8.60%, *p* < 0.001), underscoring the role of systemic adiposity in the disease. Basal metabolic rate (BMR), another critical metabolic measure, was also significantly higher in MAFLD participants (1566.09 ± 27.37 kcal/day vs. 1429.86 ± 93.44 kcal/day, *p* < 0.001), indicating elevated energy demands associated with increased lean and fat mass. These differences highlight the complex interplay between body composition and metabolic activity in MAFLD pathogenesis. Muscle mass percentage was lower in MAFLD participants (29.62 ± 3.50% vs. 33.18 ± 4.12%, *p* < 0.01), indicating a trend toward sarcopenic obesity—a condition characterized by increased fat mass and reduced muscle mass. The combination of increased fat mass and decreased muscle mass causes exacerbated insulin resistance and reduced energy expenditure, both of which are key factors in MAFLD progression, highlighting the importance of muscle preservation and fat reduction via training and dietary modifications, which may be particularly effective in mitigating MAFLD progression.

Our study’s anthropometric and metabolic results are in good agreement with regional, national, and worldwide MAFLD studies. Reduced muscle mass, systemic fat buildup, and central obesity (as determined by BMI and WC) are frequently cited as major causes of MAFLD. According to a study done in Egypt, patients with MAFLD had an average BMI of 30.8 kg/m2, which further supports the idea that obesity is a major contributing factor to MAFLD^43^. Similarly, a study conducted in Saudi Arabia discovered a high correlation between the severity of steatosis and BMI levels of more than 30–40 kg/m^2^^60^. Another study conducted in Egypt found that WC > 90 cm in males and > 80 cm in women was a significant predictor of hepatic steatosis^42^. Global evidence from a Brazilian study also supports this, which indicates that WC > 99 cm is a more significant anthropometric biomarker of central obesity and a stronger predictor than BMI alone for screening adolescents at higher risk of MAFLD^61^. The growth of adipose tissue causes systemic inflammation, which requires more energy to regulate. Cytokines like TNF-α and interleukin-6 (IL-6) play a major role in this process^47,62^. A hallmark of MAFLD is hepatic mitochondrial dysfunction, which results in inefficient ATP synthesis, requiring the body to use more substrates and produce heat instead of effectively storing energy^63,64^. Furthermore, sarcopenic obesity, which is characterized by decreased skeletal muscle mass and increased fat mass, makes insulin resistance worse and raises energy expenditure even more^59^. Additionally, oxidative stress and lipotoxicity from accumulating free fatty acids cause increased BMR, especially in the advanced stages of the disease^33^. These combined factors underscore the elevated metabolic demands in MAFLD patients.

The body composition analysis provides detailed insights into the proportion of various components in the body, including fat mass, muscle mass, bone density, and water content. It is critical for assessing overall health and diagnosing conditions like obesity, sarcopenia, or metabolic-associated fatty liver disease (MAFLD), where changes in body fat and muscle proportions play a significant role in disease progression and outcomes^59^. The fat mass and muscle mass analysis results agree with those of previous studies. MAFLD patients, in a South African study exhibited fat mass percentages exceeding 30%^65^. Systemic adiposity is strongly correlated with increased hepatic fat content and plays an important role in disease development and progression^59^. Reduced skeletal muscle mass (sarcopenia) was correlated with increased hepatic fat content^63^ and elevated visceral fat^47^ in MAFLD patients, as echoed by our study’s observations of sarcopenic obesity using the InBody 270 device. Reduced muscle mass impairs glucose metabolism and exacerbates insulin resistance, further promoting hepatic steatosis^66^. Reduced muscle mass and increased fat mass in MAFLD patients arise from chronic low-grade inflammation, insulin resistance, and mitochondrial dysfunction. Pro-inflammatory cytokines like TNF-α and IL-6, released by adipose tissue, impair muscle protein synthesis while enhancing lipogenesis, promoting both sarcopenia and fat accumulation^62^. Insulin resistance disrupts anabolic signaling to muscles and increases lipolysis, driving fatty acid deposition in the liver and adipose tissue and worsening metabolic dysfunction^33,59^. Mitochondrial inefficiency further reduces energy availability for muscle maintenance, while lipotoxicity exacerbates fat storage. Physical inactivity, often observed in MAFLD patients, compounds these effects by promoting fat deposition and muscle loss^63,64^.

It is now evident that our study’s dual-modality method, which combines TE with InBody 270, captures both liver-specific and systemic metabolic variables that contribute to MAFLD. By providing a more complex knowledge of the illness, this methodology makes risk classification and early intervention possible. Crucially, it tackles the need for scalable and reasonably priced population-level screening instruments, especially for young adults—like college students—who are frequently disregarded in MAFLD studies.

Prior studies have integrated TE with imaging methods like magnetic resonance elastography (MRE), which is thought to have a higher sensitivity than TE in identifying early fibrosis^33^. Nevertheless, MRE is still prohibitively expensive and less available in low-resource environments. Furthermore, in MAFLD research, bioimpedance analysis tools like the InBody 720 have demonstrated similar accuracy in determining body composition. The severity of MAFLD is significantly predicted by decreased skeletal muscle mass and elevated visceral fat^47^, findings echoed by our study’s observations of sarcopenic obesity. Recent research has used TE alongside bioimpedance analysis devices like InBody to measure both liver stiffness and body composition, linking adiposity, muscle mass, and visceral fat with liver health, making it especially relevant for MAFLD, where systemic metabolic dysfunction plays a key role^59^. For the staging of fibrosis in advanced MAFLD, TE, MRE, and two-dimensional shear wave elastography (2D-SWE) have demonstrated potential. MRE gives better quantification, while 2D-SWE offers advantages in spatial resolution. Because of its ease of use, TE is still crucial for preliminary screening^67^.

The regression analysis provided insights into the significant risk factors for both steatosis and MAFLD. Higher body weight (OR = 1.14, *p* = 0.000) and BMI (OR = 1.63, *p* = 0.000) were strong predictors, reflecting the critical role of general obesity in the disease. Waist circumference (OR = 1.15, *p* = 0.03) and basal metabolic rate (OR = 1.00, *p* = 0.04) further underscored the contribution of central adiposity and heightened metabolic demands. Fat mass percentage (OR = 1.16,* p* = 0.00) was significantly elevated, while reduced muscle mass percentage (OR = 0.79, *p* = 0.02) highlighted the importance of sarcopenic obesity in MAFLD progression. Short weight-loss duration (3–6 weeks) significantly increased the odds of MAFLD (OR = 1.14, *p* = 0.03), but no significant effect was seen for steatosis. Poor sleep quality (OR = 5.17, *p* = 0.002) and a sedentary lifestyle (OR = 7.69, *p* = 0.000 for steatosis and OR = 1.17, *p* = 0.000 for MAFLD) also emerged as strong predictors. Unhealthy dietary practices were associated with increased risk (OR = 4.55, *p* = 0.000 for steatosis and OR = 1.01*, p* = 0.000 for MAFLD). The family history of obesity showed borderline significance for steatosis (p = 0.05) but was not significant for MAFLD. These findings emphasize the multifactorial nature of MAFLD, driven by metabolic, anthropometric, and lifestyle factors.

Complex interactions among environmental factors, metabolism and demography, genetic variants, and gut microbiota are involved in the pathogenesis of MAFLD rather than obesity alone. This may explain why MAFLD is reported in both obese and non-obese patients^68^. The main causes of MAFLD in obese patients are systemic inflammation, insulin resistance, and extra visceral fat, which raises the risk of fibrosis and increases hepatic fat deposition. Type 2 diabetes and hypertension are metabolic comorbidities that obese people frequently have, making their disease progression more aggressive^69^. However, while having a normal BMI, non-obese MAFLD (lean MAFLD) is characterized by mild metabolic abnormalities such as visceral fat accumulation, sarcopenia, and insulin resistance^70^. Genetic factors, such as patatin-like phospholipase domain-containing 3 genes (PNPLA3) polymorphisms and glucokinase regulatory protein gene Polymorphisms (GCKRP)^71^, and environmental influences like poor diet contribute significantly. While non-obese patients may appear metabolically healthier, both obese and non-obese MAFLD patients are at risk of disease progression, but non-obese individuals may experience delayed diagnosis due to a lack of overt risk factors causing advanced liver disease and cardiovascular complications^13^.

Egypt, a Middle Eastern nation with a population exceeding 105 million in 2024, has nearly 60% of the population contributing to the country′s rising obesity pandemic^43^. This is indicative of the combined burden of obesity and malnutrition that is prevalent in many low- and middle-income nations because of a move away from traditional diets and toward processed high-calorie meals. Before 2024, Egypt′s youth obesity rate was 32%, placing it seventh in the world. However, by 2024, the country′s overall obesity rate had increased to 35.7%, placing it 28th internationally, a significant increase from 31.3% in 2011-2012, underscoring the growing public health concern^72^. Despite being frequently disregarded, metabolic-associated fatty liver disease (MAFLD) is becoming more prevalent in young adults and places an enormous burden on healthcare systems^13^. The necessity for focused public health interventions is highlighted by the low level of public knowledge of MAFLD, with only 37% of Egyptians identifying obesity and metabolic dysfunction as significant risk factors^43^. High rates of obesity in Egypt are a contributing factor to MAFLD, with central obesity resulting in hepatic steatosis and visceral fat accumulation^43^. Sedentary lifestyles, diets high in unhealthy fats and processed carbohydrates, and metabolic dysfunctions such as insulin resistance, dyslipidemia, and type 2 diabetes are all contributing factors^19,43^. Even in similar environmental circumstances, genetic predispositions such as glucokinase regulatory protein (GCKR) polymorphisms, transmembrane 6 superfamily member 2 (TM6SF2), and patatin-like phospholipase domain-containing 3 (PNPLA3) further increase the risk of MAFLD^34,73^.

Globally, obesity and sedentary lifestyles are the dominant risk factors for metabolic-associated fatty liver disease (MAFLD), with prevalence rates as high as 25–30% in some populations. Urbanization and increased sedentary behaviors with poor sleep quality further exacerbate hepatic fat storage and disease progression^74,75^. By encouraging lipogenesis and decreasing fat export, insulin resistance contributes significantly to the pathophysiology of MAFLD. When it co-occurs with type 2 diabetes, the risk of progressive fibrosis and unfavorable outcomes is increased^76^. Hepatic fat deposition and oxidative stress are greatly influenced by dietary factors, especially in urbanized areas, such as the excessive intake of fructose, trans fats, and processed foods^77^. As a reflection of the global variety of MAFLD etiology, non-obese or “lean” MAFLD is more common in Asia, where genetic variables, including PNPLA3 polymorphisms, and high-carb diets greatly contribute to its development^36^. Smoking and moderate alcohol use can aggravate hepatic inflammation and fibrosis, increasing the disease burden in many groups, even if excessive alcohol consumption is not a fundamental cause of MAFLD. Furthermore, low- and middle-income nations are disproportionately affected by sociocultural and economic variables, such as rapid urbanization, economic transformations, and restricted access to healthcare, which contribute to the rising prevalence and advancement of MAFLD^13^. Dysbiosis, disruption or imbalance in the gut flora, is linked to obesity, type 2 diabetes, fatty liver disease, inflammatory bowel disorders, several types of cancer, as well as the development and progression of MAFLD. Dysbiosis of the gut microbiota produces pro-inflammatory compounds like lipopolysaccharides (LPS), which cause inflammation in the liver. Additionally, it interferes with the metabolism of bile acids, which leads to inflammation and hepatic fat buildup. Furthermore, modifications to the gut microbiota can influence how short-chain fatty acids (SCFAs) are produced, which can impact metabolic functions. Thus, treating gut dysbiosis provides possible therapeutic approaches to enhance MAFLD management.^46^.

Recent studies have further elucidated the complex interplay between metabolic dysfunction and MAFLD, highlighting the importance of early detection and intervention. For instance, research by^78^ demonstrated that insulin resistance and dyslipidemia are key drivers of MAFLD progression, particularly in younger populations, underscoring the need for targeted metabolic screening in at-risk groups. Similarly,^79^ identified a strong association between MAFLD and cardiovascular risk factors, suggesting that MAFLD may serve as a marker for broader metabolic syndrome. Additionally,^80^ explored the role of gut microbiota in MAFLD pathogenesis, revealing that dysbiosis contributes to hepatic inflammation and fibrosis, which aligns with our findings on the multifactorial nature of MAFLD. Furthermore,^81^ emphasized the impact of lifestyle factors, such as sedentary behavior and poor dietary habits, on MAFLD development, reinforcing the importance of lifestyle interventions in disease prevention. The study by^82^ highlighted the utility of non-invasive diagnostic tools, such as transient elastography, in identifying early-stage MAFLD, which complements our methodological approach. Lastly,^83^ investigated the genetic predispositions to MAFLD, providing insights into why certain individuals are more susceptible to metabolic dysfunction even in the absence of traditional risk factors. Collectively, these studies underscore the multifactorial nature of MAFLD and the need for a holistic approach to its management, integrating metabolic, genetic, and lifestyle factors.

Future research should focus on longitudinal designs to better understand disease progression and explore the integration of advanced diagnostics, such as MRE and biomarkers, in routine screening protocols to improve accuracy and predictive value. account for all possible confounders, such as genetic predispositions or other health conditions will also give a better insight. Additionally, public health campaigns emphasizing the importance of early screening and lifestyle interventions are crucial in combating the rising prevalence of MAFLD among young adults.

This study has several limitations. First, the cross-sectional design prevents the establishment of causal relationships between risk factors and MAFLD; future cohort studies are needed to explore temporal relationships and disease progression. Second, the sample was limited to medical students, which may not be representative of the general population, potentially introducing selection bias. Third, measurement variability in transient elastography (TE) and InBody 270 assessments could have occurred due to factors such as hydration status or recent food intake. Fourth, the absence of longitudinal data limits our ability to track MAFLD progression and long-term outcomes. Finally, reliance on self-reported data for dietary habits, sleep quality, and physical activity may have introduced recall bias, potentially affecting the accuracy of lifestyle-related findings. Future longitudinal studies involving more diverse populations are needed to confirm these associations and better understand disease progression over time.

## Conclusion

The findings of this study highlight the significant frequency of MAFLD in a cohort of young, seemingly healthy medical students, with several key risk factors identified through comprehensive assessments. Elevated body mass index (BMI), central obesity, poor sleep quality, sedentary lifestyle, and unhealthy dietary patterns emerged as the most prominent contributors to MAFLD. These factors emphasize the importance of addressing lifestyle modifications early, particularly in younger populations, to prevent the progression of MAFLD. The results underscore the need for public health interventions aimed at improving awareness and early detection of MAFLD.

## Data Availability

All relevant data are included in this published article.

## Abbreviations

BMR: Basal metabolic rate
BMI: Body mass index
CAP scan: Controlled attenuated parameter scan
F: Fibrosis
InBody: Bioelectrical impedance analysis device (InBody 270)
LPS: Lipopolysaccharides
LSM: Liver stiffness measurement
MAFLD: Metabolic-associated fatty liver disease
MetS: Metabolic syndrome
NASH: Non-alcoholic steatohepatitis
NAFLD: Non-alcoholic fatty liver disease
OR: Odds ratio
PNPLA3: Patatin-like phospholipase domain-containing protein 3
SCFAs: Short-chain fatty acids
SD: Standard deviation
T2D: Type 2 diabetes
TE: Transient elastography
TM6SF2: Transmembrane 6 superfamily member 2
WC: Waist circumference
